# Liver Decompensation as Late Complication in HCC Patients with Long-Term Response following Selective Internal Radiation Therapy

**DOI:** 10.3390/cancers13215427

**Published:** 2021-10-29

**Authors:** Diederick J. van Doorn, Pim Hendriks, Mark C. Burgmans, Daphne D. D. Rietbergen, Minneke J. Coenraad, Otto M. van Delden, Roel J. Bennink, Tim A. Labeur, Heinz-Josef Klümpen, Ferry A. L. M. Eskens, Adriaan Moelker, Erik Vegt, Dave Sprengers, Nahid Mostafavi, Jan Ijzermans, R. Bart Takkenberg

**Affiliations:** 1 Department of Gastroenterology and Hepatology, Amsterdam UMC, University of Amsterdam, Meibergdreef 9, 1105 AZ Amsterdam, The Netherlands; d.j.vandoorn@amsterdamumc.nl (D.J.v.D.); t.a.labeur@amsterdamumc.nl (T.A.L.); 2 Department of Radiology, Leiden University Medical Center, Albinusdreef 2, 2333 ZA Leiden, The Netherlands; p.hendriks@lumc.nl (P.H.); m.c.burgmans@lumc.nl (M.C.B.); 3 Department of Radiology and Nuclear Medicine, Leiden University Medical Center, Albinusdreef 2, 2333 ZA Leiden, The Netherlands; d.d.d.rietbergen@lumc.nl; 4 Department of Gastroenterology and Hepatology, Leiden University Medical Center, Albinusdreef 2, 2333 ZA Leiden, The Netherlands; m.j.coenraad@lumc.nl; 5 Department of Radiology and Nuclear Medicine, Amsterdam UMC, University of Amsterdam, Meibergdreef 9, 1105 AZ Amsterdam, The Netherlands; o.m.vandelden@amsterdamumc.nl (O.M.v.D.); r.bennink@amsterdamumc.nl (R.J.B.); 6 Department of Medical Oncology, Amsterdam UMC, University of Amsterdam, De Boelelaan 1105, 1081 HV Amsterdam, The Netherlands; h.klumpen@amsterdamumc.nl; 7 Department of Medical Oncology, Erasmus MC Cancer Institute, Doctor Molewaterplein 40, 3015 GD Rotterdam, The Netherlands; f.eskens@erasmusmc.nl; 8 Department of Radiology and Nuclear Medicine, Erasmus MC University Medical Center Rotterdam, Doctor Molewaterplein 40, 3015 GD Rotterdam, The Netherlands; a.moelker@erasmusmc.nl (A.M.); e.vegt@erasmusmc.nl (E.V.); 9 Department of Gastroenterology and Hepatology, Erasmus MC University Medical Center, Doctor Molewaterplein 40, 3015 GD Rotterdam, The Netherlands; d.sprengers@erasmusmc.nl; 10 Department of Gastroenterology and Hepatology, Biostatistical Unit, Amsterdam UMC, University of Amsterdam, Meibergdreef 9, 1105 AZ Amsterdam, The Netherlands; n.mostafavi@amsterdamumc.nl; 11 Department of Surgery, Erasmus MC University Medical Center Rotterdam, Doctor Molewaterplein 40, 3015 GD Rotterdam, The Netherlands; j.ijzermans@erasmusmc.nl

**Keywords:** hepatocellular carcinoma, selective internal radiation therapy, long-term response, liver decompensation, overall survival

## Abstract

**Simple Summary:**

Hepatocellular carcinoma (HCC) is one of the deadliest forms of cancer. Selective internal radiation therapy (SIRT) is one of the therapeutic options for treatment of advanced HCC. Studies show that SIRT has a high objective response rate, but lack of survival benefit when compared to different treatment modalities. We hypothesized that this is due to potential damage in healthy liver parenchyma as a side-effect of SIRT, resulting in functional changes to the liver. This can ultimately result in liver decompensation and potentially death. The aim of this retrospective study was to assess long-term liver-related complications after SIRT in patients with HCC. We analyzed patients who underwent SIRT and found that liver decompensation occurred more often after SIRT when compared to sorafenib. However, careful patient selection may result in a survival benefit after SIRT when compared to other treatments. The ABLI score may be a valuable prognostic score for selecting patients.

**Abstract:**

Selective internal radiation therapy (SIRT) is used as a treatment for hepatocellular carcinoma (HCC). The aim of this study was to assess long-term liver-related complications of SIRT in patients who had not developed radioembolization-induced liver disease (REILD). The primary outcome was the percentage of patients without REILD that developed Child-Pugh (CP) ≥ B7 liver decompensation after SIRT. The secondary outcomes were overall survival (OS) and tumor response. These data were compared with a matched cohort of patients treated with sorafenib. Eighty-five patients were included, of whom 16 developed REILD. Of the remaining 69 patients, 38 developed liver decompensation CP ≥ B7. The median OS was 18 months. In patients without REILD, the median OS in patients with CP ≥ B7 was significantly shorter compared to those without CP ≥ B7; 16 vs. 31 months. In the case-matched analysis, the median OS was significantly longer in SIRT-treated patients; 16 vs. 8 months in sorafenib. Liver decompensation CP ≥ B7 occurred significantly more in SIRT when compared to sorafenib; 62% vs. 27%. The ALBI score was an independent predictor of liver decompensation (OR 0.07) and OS (HR 2.83). After SIRT, liver decompensation CP ≥ B7 often developed as a late complication in HCC patients and was associated with a shorter OS. The ALBI score was predictive of CP ≥ B7 liver decompensation and the OS, and this may be a valuable marker for patient selection for SIRT.

## 1. Introduction

Hepatocellular carcinoma (HCC) is the sixth most common type of cancer worldwide [1]. Staging of HCC follows the Barcelona clinic liver cancer (BCLC) staging system. In this classification, the disease is divided into (very) early-stage (BCLC 0/A), intermediate-stage (BCLC B), advanced-stage (BCLC C) and end-stage disease (BCLC D) [2]. The standard of care for patients with BCLC stage B HCC is transarterial chemoembolization (TACE). Guidelines recommend systemic therapy in patients with BCLC stage C HCC [3]. International guidelines advise that selective internal radiotherapy (SIRT) may be considered for BCLC stage B HCC in patients in whom TACE is not an option (i.e., beyond TACE) or BCLC stage C HCC with macrovascular invasion and without distant metastases [4,5]. SIRT is a form of brachytherapy, which uses microspheres loaded with beta-emitting isotopes, usually yttrium-90 (^90^Y) or in alternative cases holmium-166 (^166^Ho) [6]. These microspheres are delivered to the tumor using intra-arterial injection in (branches of) the hepatic artery. Although proven safe, SIRT has not yet reached a well-defined place in the treatment algorithm of HCC [4,5]. Two randomized controlled trials, SIRveNIB and SARAH, showed that SIRT has a higher objective response rate compared to sorafenib, but with no impact on overall survival (OS) [7,8]. These studies showed a median OS of 17 months for patients with BCLC stage B HCC and 10 to 12 months for patients with BCLC stage C HCC treated with SIRT [9,10,11,12]. Furthermore, another trial (SORAMIC) showed that the addition of SIRT to sorafenib did not lead to a significant improvement in OS, with a median OS of 12 months in the SIRT plus sorafenib arm, compared to 11 months in the sorafenib monotherapy arm [13].

One severe complication of SIRT is radioembolization-induced liver disease (REILD), which is defined as a symptomatic deterioration of the liver function, developing between two weeks and four months after SIRT, in the absence of tumor progression or biliary tract obstruction. In previous studies, the incidence of REILD ranged from 0 to 31% [14]. There are only a few small studies that have reported on the long-term liver-related outcome after SIRT. These studies revealed that SIRT was associated with hepatic volume changes, liver fibrosis, portal hypertension and an increase in splenic volume. These effects usually have their onset 4–20 weeks after SIRT and continue to develop over time [15,16]. The damage to non-tumorous liver parenchyma resulting in loss of liver function, progression of cirrhosis or liver failure might negatively impact survival outcomes in patients with HCC.

In this study, we aimed to assess the long-term liver-related complications (deterioration of liver function, progression of liver cirrhosis or portal hypertension) in patients with BCLC stage B HCC who were not eligible for TACE or patients with BCLC stage C HCC confined to the liver with macrovascular invasion, who were treated with SIRT and did not develop REILD. We aimed to identify predictors of these liver-related complications.

## 2. Materials and Methods

### 2.1. Study Population

All patients diagnosed with HCC according to the EASL guidelines, and considered eligible for treatment with SIRT by the local multidisciplinary tumor board for primary liver cancer at three university medical centers (Amsterdam University Medical Center (Amsterdam UMC), Leiden University Medical Center (LUMC) and Erasmus Medical Center Rotterdam (Erasmus MC)) between 2011 and 2019 were included in the study screening. Patients were diagnosed in accordance with international guidelines by radiological criteria, or when cirrhosis was not present, by histology [4,5]. Diagnosis was made by multi-phase computed tomography (CT) or dynamic contrast-enhanced magnetic resonance imaging (MRI) and all patients were discussed at a multidisciplinary meeting with surgeons, medical oncologists, gastroenterologists, (interventional) radiologists and nuclear medicine physicians.

**Figure 1:**
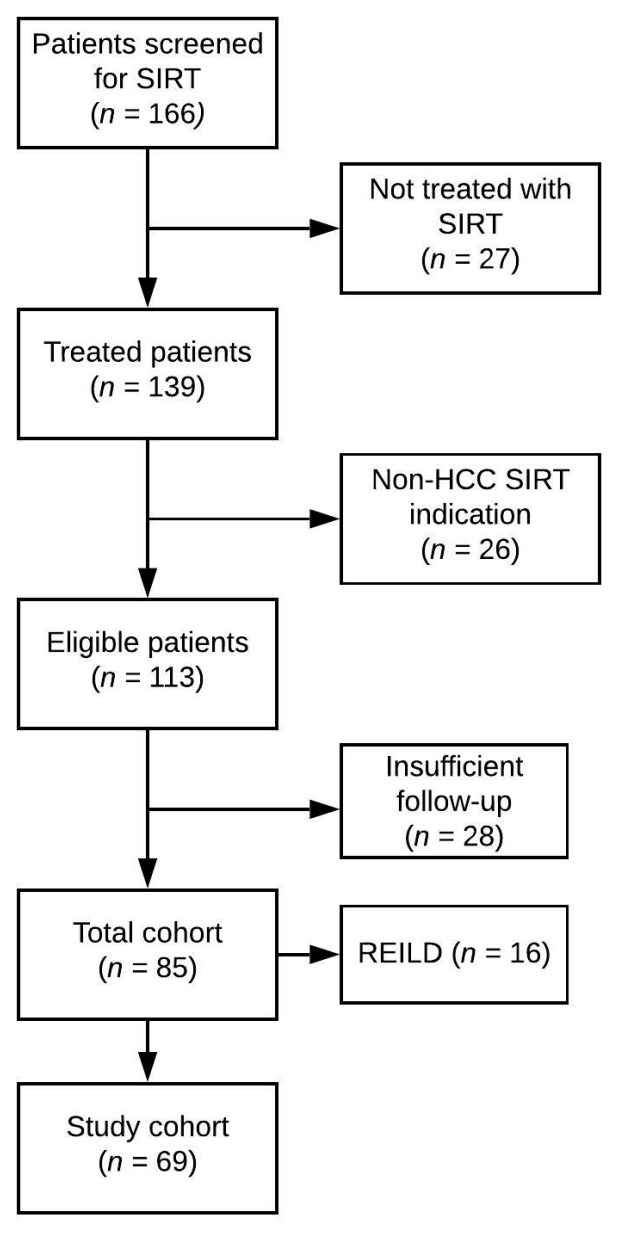
Inclusion flowchart. SIRT: Selective Internal Radiation Therapy; Non-HCC: Patients who underwent SIRT for clinical indications other than hepatocellular carcinoma; REILD: Radioembolization-induced liver disease.

The aim of this study was to assess the long-term liver-related complications in patients receiving SIRT who did not develop REILD, and to identify predictors of the treatment outcome. Patients were divided in two different cohorts. The first cohort included all patients treated with SIRT (total cohort). In the second cohort (study cohort), patients who died or developed REILD within four months after SIRT were excluded (shown in Figure 1).

The data were collected from electronic patient records after permission was gained from the local ethics committees of each participating center to waive the necessity of written informed consent for this retrospective study.

Case-matched cohort analysis was performed with a cohort (*n* = 300) of patients treated with sorafenib between January 2007 and December 2016 in the Amsterdam UMC and Erasmus MC. These patients were analyzed in a retrospective study, which was published earlier [17].

### 2.2. Data Collection

Patients were identified using the code for pre-SIRT workup, which could be captured from the electronic patient record (EPD). In the case of consecutive SIRT sessions in one patient, the data were collected from the first session onwards. Patients’ clinical, radiological, nuclear medicine and laboratory data were manually extracted from their medical files, as were all relevant SIRT data. The collected data consisted of patient baseline characteristics, disease etiology and previous treatments. Baseline laboratory results, together with imaging, clinical data and tumor characteristics, were obtained prior to SIRT. Child-Pugh (CP), model for end-stage liver disease (MELD) (pre-2016) and albumin-bilirubin (ALBI) scores were calculated for each patient.

Treatment details of SIRT such as the dosage, segments treated, tumor response and treatment-related adverse events (AEs) were collected. Follow-up data consisted of laboratory assessments and imaging results obtained six weeks, three months, six months, nine months and one year after SIRT. In the case of survival longer than one year, the liver-related outcome was observed until decompensation, loss to follow-up or death. All data were anonymized, coded and entered into an online database management system (Castor EDC).

### 2.3. Outcome Measures

The primary outcome was the percentage of patients without REILD that developed CP score ≥ B7 liver decompensation four months or more after SIRT. The Common Terminology Criteria for Adverse Events (CTCAE) recently released version 4.0 of its definitions, including chronic hepatotoxicity.

Even though it is a complete and precise criterion describing hepatotoxicity, it lacks a cumulative scoring system. We, therefore, chose CP ≥ B7 as the endpoint as it correlates most with CTCAE grade ≥ 3 liver toxicity and due to its wide application by clinicians.

REILD was defined as a symptomatic post-radioembolization deterioration in the ability of the liver to maintain its (normal or preprocedural) synthetic, excretory and detoxifying functions, characterized by the onset of jaundice, new onset or increase in ascites, hyperbilirubinemia and hypoalbuminemia developing at least two weeks and no later than four months after RE, which could not be explained by either tumor progression or biliary tract obstruction [12]. Long-term liver-related complications were defined as clinical or biochemical presentation of liver decompensation that occurred at the first follow-up after the defined four months after SIRT. Liver decompensation was scored according to the CP classification, whereby decompensation was considered in case of a CP B7 or higher when initially CP A. Other scoring and used definitions of criteria are summarized in the supplementary text.

The secondary outcomes were OS, tumor response and time to progression (TTP). For OS, the date of the first SIRT until the date of death for any reason was used, or censored on the last known date to be alive. TTP was defined as the date of SIRT until radiological disease progression. Patients without progression were censored on the last date of follow-up. Radiological response evaluation was performed every three months using the Response Evaluation Criteria in Solid Tumors (RECIST 1.1) [18]. For analysis for the cause of death, patients were categorized into four groups: tumor-related death, liver-related death, combined (i.e., liver plus tumor-related) cause or other/unknown cause. Death was considered tumor-related in the case of a progressive disease without the occurrence of liver decompensation. Death was considered liver-related in the case of liver decompensation without documentation of tumor progression, and combined in the case of liver decompensation and progressive disease. Patients’ last available follow-up results were clustered and analyzed.

For the comparison of OS between the SIRT-treated patients and the matched sorafenib-treated patients, two separate propensity-matched analyses were done: one for all patients after treatment (including patients who developed REILD) and one for all patients who received a minimum of four months of sorafenib versus patients who did not develop REILD. For comparison between SIRT and sorafenib in terms of liver decompensation, grade 3-4 liver toxicity according to the CTCAE in the sorafenib cohort was compared with the occurrence of liver decompensation (CP ≥ B7) in the SIRT cohort. Patient and matching characteristics of the matched cohorts are shown in supplementary Tables S1–S4.

### 2.4. Statistical Analysis

Continuous variables were reported as the mean with standard deviation (SD), or where appropriate, as the median with interquartile range. Categorical variables were reported as absolute values with percentages. For comparison of baseline and follow-up data, a paired *T*-test was used in the case of normally distributed data. In the case of non-normally distributed data, a Wilcoxon signed rank test was used. For comparison between groups, a Mann-Whitney U-test was used when appropriate. When comparing categorical data, 2 × 2 contingency tables were created and analyzed with the appropriate test, which was either the Pearson’s chi-square or Fisher’s exact test.

For OS and TTP estimates, comparison and figures, the Kaplan-Meier method was used and log-rank tests were performed, and Cox proportional hazard regression analysis was used to calculate hazard ratios (HRs). Cox proportional hazard regression and logistic regression were used to assess the association between different baseline variables/predictors and survival or liver decompensation, respectively.

For comparison of OS and occurrence of liver decompensation between patients treated with sorafenib and SIRT, propensity score matching was performed to create maximally balanced groups. For OS, the total cohort of SIRT patients was matched with sorafenib patients. For long-term liver-related complications, the long-term survival cohort of SIRT patients was matched with sorafenib patients treated with sorafenib for at least four months. Matching was done using the optimal method, focusing on minimizing the average absolute distance between all pairs based on sex, age, cirrhosis, CP score, portal hypertension, liver-confined disease and BCLC classification, in a ratio of 1:1. Standardized mean differences (SMD) of each covariate were used to determine whether matching improved balance. Matching was performed separately for each outcome (OS in the total cohort of treated patients, and occurrence of liver decompensation), for optimal comparability.

A two-sided *p* value < 0.05 was considered statically significant. Statistical analyses were performed using SPSS statistics (version 26.0; IBM Corp. Armonk, NY: IBM Corp.). Propensity analyses were performed using the MatchIt package in R (version 3.6.1, https://cran.r-project.org/ (accessed on 15 August 2021)).

## 3. Results

### 3.1. Patient Characteristics

For this analysis, 85 patients treated with SIRT between June 2011 and March 2019 were identified, who comprised the total cohort. Sixteen patients (19%) developed significant liver toxicity within four months after SIRT, meeting the REILD criteria. No patient developed decompensation due to tumor progression or biliary tract obstruction. The remaining 69 (81%) patients were included in the study cohort. The baseline characteristics for both cohorts, as well as the tumor and previous treatment characteristics, are summarized in Table 1.

### 3.2. Total Cohort (All Patients Who Received SIRT)

For the total cohort of 85 patients, the most common HCC etiology was alcohol (44%), followed by hepatitis C virus infection (19%) and hepatitis B virus infection (12%). Sixty-two patients (73%) had underlying liver cirrhosis, of whom 59 patients (95%) had CP A5 or A6. Forty-two patients (49%) had portal hypertension at the baseline. Nine patients (13%) had ascites on imaging, of whom three patients (33%) had clinically relevant ascites. At the baseline, patients had a median MELD score of 8 and a mean ALBI score of −2.7. Translated into ALBI grades, this accounted for 55 patients (65%) with ALBI grade 1 and 30 patients (35%) with ALBI grade 2.

At the time of SIRT, 34 patients (40%) had received any other prior treatment, mostly TACE (19%), radiofrequency ablation or microwave ablation (17%). At the baseline, 52 patients (61%) had BCLC stage B HCC. Of the 85 patients who had been treated with SIRT, 8 had received whole liver treatment, 63 lobar treatment (of whom 19 with an additional segmental treatment in the contralateral lobe) and 14 segmental treatment. Patients treated with SIRT received a median activity of 1930 MBq ^90^Y (range 500–7200 MBq). There was no relation between the total dose and long-term complication rate.

### 3.3. Study Cohort (Patients Who Received SIRT Who Did Not Develop REILD)

#### 3.3.1. Development of Liver Decompensation during Follow-Up

**Figure 2:**
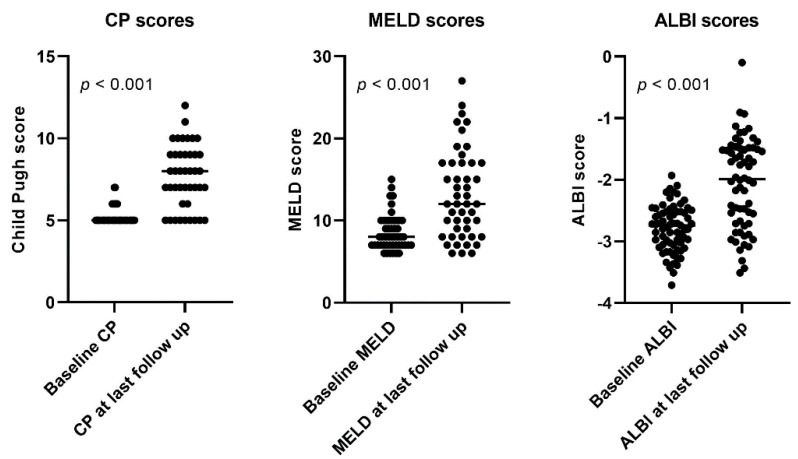
Liver function scores at baseline compared to last follow-up after SIRT in the study cohort. ALBI: albumin-bilirubin; CP: Child Pugh; MELD: model for end-stage liver disease.

In the study cohort, after a median follow-up of 30 months (95% confidence interval (CI) 18–41), 38/69 patients (55%) developed liver decompensation CP ≥ B7 (shown in Table 2). From these 38 patients, 23 (61%) had hyperbilirubinemia, 31 (82%) had hypoalbuminemia, 7 (18%) experienced an episode of hepatic encephalopathy and 35 patients (92%) had newly onset ascites, of whom 30 (86%) required intervention. At the end of follow-up, for the CP score, a signed-rank test indicated that the follow-up CP (median = 7) was significantly higher than at the baseline (median = 5) (Z = 30.0, *p* < 0.001). For the MELD score, a signed-rank test indicated that the follow-up MELD (median = 12) was significantly higher than at the baseline (median = 8) (Z = 26.0, *p* < 0.001). For the ALBI score, a paired *T*-test showed a significant increase of the ALBI score at the follow-up when compared to the baseline (baseline mean = −2.8, SD 0.37; follow-up mean = −2.1, SD: 0.73; conditions t(63) = −9.6, *p* < 0.001) (shown in Figure 2).

The presence of liver cirrhosis at the baseline was significantly associated with liver decompensation at the last follow-up after SIRT (odds ratio (OR) of 4.0 (95% CI 1.2–13.2, *p* = 0.018)) (shown in Table 3). Furthermore, a lower ALBI score at the baseline was also significantly associated with a better outcome, with an OR of 0.074 per point of absolute increase (i.e., more negative ALBI score) (95% CI 0.012–0.475, *p* = 0.006) (shown in Table 3). After multivariate analysis, the ALBI score remained an independent predictor of liver decompensation at the last follow-up (OR of 0.114 (95% CI 0.016–0.824, *p* = 0.031)), making it the only predictor that showed a significant correlation with liver decompensation at last follow-up.

#### 3.3.2. Treatment and Tumor Response

During follow-up, 46 patients (67%) had tumor progression. At the last follow-up, 5 patients had a complete response (7%), 13 (19%) had a partial response, 13 (19%) had a stable disease and 30 (43%) had a progressive disease (shown in Table 2). In six patients (9%) the response could not be determined, and in two patients (3%) data were missing. The median TTP was not significantly different between patients who had liver decompensation at the last follow-up (six months (95% CI 4.6–7.4)) and patients who did not decompose at the last follow-up (six months 95% CI 4.4–7.6) (*p* = 0.899). In addition, there was no difference in the ^90^Y activity between patients who decomposed at the last follow-up and those who did not (*p* = 0.820).

### 3.4. Overall Survival

**Figure 3:**
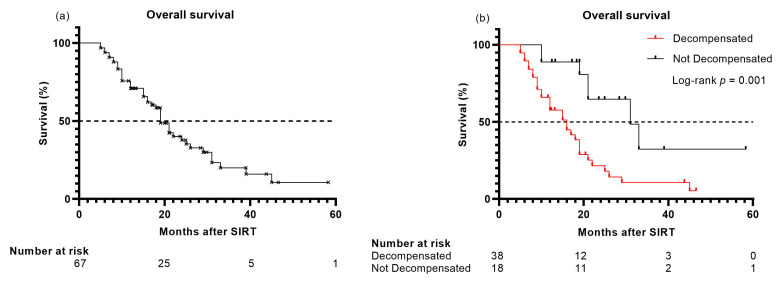
(**a**) Overall survival (study cohort); and (**b**) Overall survival (study cohort) in patients who developed liver decompensation compared with patients who did not develop liver decompensation at last follow-up.

The median OS in the total cohort of SIRT-treated patients was 18 months (95% CI 14–22). In the study cohort, 45/69 patients (65%) had died after a median follow-up of 30 months (95% CI 18–41). The median OS in this study cohort was 19 months (95% CI 17–21) (shown in Figure 3a). Of these 45 patients, four (9%) died due to tumor-related complications without any signs of liver dysfunction. Ten patients (22%) died due to liver-related complications, such as liver failure or varices bleeding, without signs of tumor progression at the time of the last follow-up. Eighteen patients (40%) died with tumor progression in combination with liver decompensation. There was no significant difference in OS between patients who died due to liver-related complications (median OS 12 months, 95% CI 5–19) and patients who died with tumor progression at the end of the follow-up (median OS 15 months (95% CI 7−22); *p* = 0.752).

For patients who developed liver decompensation (*n* = 38), the median OS was 16 months (95% CI 11−21), which was significantly shorter compared to the OS in patients who did not develop liver decompensation (median OS 31 months (95% CI 19−43); *p* = 0.001) (shown in Figure 3b). The ALBI score was an independent predictor of OS (HR 2.83; 95% CI 1.43−5.60; *p* = 0.003).

### 3.5. Propensity-Matched Analysis of Survival in SIRT Versus Sorafenib

**Figure 4:**
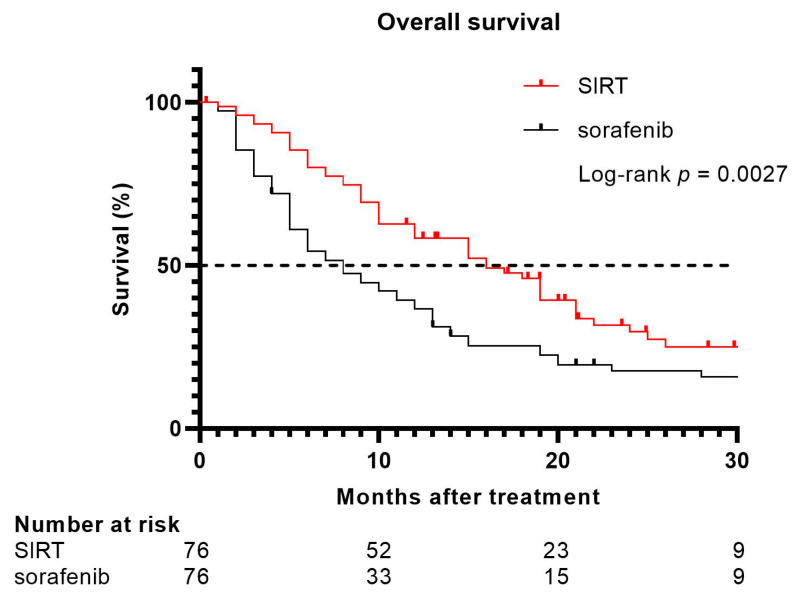
Overall survival in all patients treated with SIRT matched with patients treated with sorafenib.

In total, 76 matched patients were included in both groups. The patient characteristics of the matched cohorts are shown in supplementary Tables S1–S4. After a median follow-up of 30 months from their start of treatment, SIRT-treated patients showed a significantly longer median OS (16 months; 95% CI 12–21) compared to patients treated with sorafenib (median OS eight months; 95% CI 6–12; *p* = 0.0027) (shown in Figure 4). Patients who underwent SIRT showed a median progression-free survival (PFS) of nine months (95% CI 6–12). Patients who were treated with sorafenib had a shorter PFS (six months; 95% CI 2–9). However, the difference in PFS was not significant when comparing the two treatment modalities (*p* = 0.14).

### 3.6. Long-Term Liver Decompensation: SIRT Matched with Sorafenib

**Figure 5:**
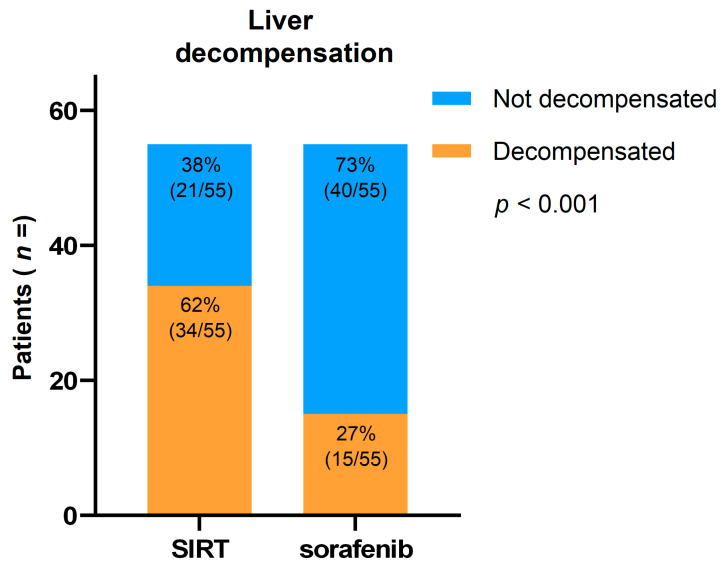
Number of patients with liver decompensation at last follow-up, where SIRT is compared with a matched cohort of patients treated with sorafenib.

In unmatched cohorts, the occurrence of liver decompensation in patients treated with SIRT was 55%, and in patients treated with sorafenib for at least four consecutive months was 22%. Secondary analysis was performed on the matched cohorts. Patients treated with SIRT and sorafenib were matched for comparison of the occurrence of liver decompensation. Ten patients with missing values in the SIRT cohort were excluded from this analysis, which showed higher occurrence of liver decompensation in patients treated with SIRT. Liver decompensation occurred significantly more often in patients who were treated with SIRT (62%) compared to patients treated with sorafenib (27%) (35% difference; 95% CI 0.28–0.57; *p* < 0.001; shown in Figure 5).

## 4. Discussion

This study aimed to assess the long-term liver-related complications in patients with BCLC B and C HCC who were treated with SIRT and did not develop REILD within the first four months after SIRT. We hypothesized that despite better response rates with fewer short-term side effects, the long-term liver-related toxicity of SIRT would be more pronounced when compared to that of other treatment modalities such as sorafenib. This would then explain the lack of benefit for OS as reported previously [11,12,13]. In this retrospective analysis, we found that in patients who did not develop REILD after SIRT, still more than half (55%) developed liver decompensation CP ≥ B7. Decompensation was associated with a significantly shorter OS compared to patients with preserved liver function. Liver decompensation occurred significantly more often after SIRT compared to matched patients treated with sorafenib (SIRT 62% vs. sorafenib 27%). Despite these liver-related complications, OS after SIRT was significantly longer than after sorafenib when patients did not develop REILD. The ALBI score was predictive of long-term liver-related complications as well as OS.

More patients experienced liver decompensation after SIRT during long-term follow-up as compared to patients treated with sorafenib. Several studies showed that the function of treated liver parenchyma declined after SIRT, with a variable compensatory increase of liver volume in the non-treated part [19]. However, a recent study from our group showed that this volume-increased non-treated part was unable to compensate for the loss of liver function in the treated part [19]. The most probable explanation would be that the untreated liver segments possess insufficient reserve capacity to (fully) compensate for the loss of function from treated liver segments. This phenomenon would be even greater in patients with HCC, who mostly have liver cirrhosis, and thus have a worse regenerative capacity of the liver to begin with [20]. It could also imply that SIRT results in significant damage to the non-tumorous liver parenchyma in the treated (and also untreated) regions. REILD is acknowledged as an acute liver injury due to radiation, and yet our current findings and prior studies have shown that delayed effects of SIRT may result in long-term liver toxicity as well. It seems that the definition of REILD falls short regarding the timeframe it has to occur in. It is clear that REILD is a problem where hepatocyte regeneration no longer occurs as a collateral effect of radiation, up to a point where liver decompensation develops. This can happen within four months, but also beyond that timeframe. This might explain why patients who have better liver function at the baseline have a better outcome. In this study, the ALBI score was identified as an independent predictor at the baseline, which showed a correlation with long-term liver decompensation as well as OS. ALBI was first described in 2015, and offers a simple and objective method of assessing the liver function in patients with HCC [21]. The model has been validated in more than 46,000 patients [22]. Lower ALBI scores were prognostic of less liver decompensation and better OS, independent of Child-Pugh scores. This could be a valuable model by which to select patients who are candidates for SIRT in the future. A recent study endorses the finding that SIRT can have a negative impact on liver function and correlates to the ALBI score [23]. Therefore, further analyses of cut-off values for the ALBI score are recommended, as well as validation in a larger cohort.

This study showed that the OS in patients treated with SIRT was significantly longer than in patients treated with sorafenib. This is in contrast with the current literature, which does not report a survival benefit of SIRT compared to systemic treatment in large phase-three trials [7,8]. However, earlier mentioned studies do suggest a survival benefit for patients treated with SIRT [9,10,11,12]. The SARAH trial gave us the inspiration to further investigate the relationship of dosage of ^90^Y, survival and response for patients treated with SIRT [24]. This study shows that optimization of the tumor dosage of ^90^Y is associated with better overall survival. In line with this study, more recent hypotheses are focusing on patient and tumor characteristics, and adjusting the treatment in accordance (i.e., higher dosage of ^90^Y on smaller segments whilst sparing liver function due to less damage to healthy liver parenchyma), which can lead to a better response and survival benefit.

Still, the most obvious explanation for the difference between the outcome of the phase-three trials and our study could be that, in this study, patients with less advanced disease were included. In the SARAH trial, with only 28% of patients with BCLC B HCC and 68% with BCLC stage C HCC, the median OS after SIRT was eight months. In this current analysis, 54% of patients had BCLC B HCC, which likewise explains the better results. In the SIRveNIB study 51% of patients had BCLC C HCC, but still the median OS after SIRT only reached 8.8 months. Another difference between the studies that could explain the difference in OS is that, for example, in the SIRveNIB study, patients with underlying hepatitis B (51%) or C (14%) as the etiology for HCC were dominant, whereas our study involved mostly alcohol-related HCCs. Furthermore, although the objective response rates after SIRT in the SARAH and SIRveNIB trials were significantly higher compared to those after sorafenib, the observation that the OS was not different could also be the result of the here described long-term complications after SIRT. Although CP classification was not different between the SIRT and sorafenib cohorts, it is plausible that in this real-life cohort, patients with better-preserved liver function were included, which could be reflected in a lower ALBI score, and therefore, longer survival. Unfortunately, the SARAH and SIRveNIB trials did not report ALBI scores. Of course, comparing SIRT with sorafenib retrospectively is prone to various kinds of bias, primarily related to patient selection. Matching patients for tumor specific criteria such as BCLC may compensate for some of these differences, but will not compensate for all potential differences between the two groups.

This study also has several limitations, most importantly its retrospective design, with some inherent drawbacks, such as the lack of availability of important data. Another limitation is the observer or investigator bias that could be the case in radiological assessment of tumor response. This is particularly difficult in patients treated with SIRT since the radiation-induced changes in liver parenchyma impede objective response evaluation according to tumor size or contrast-enhancement. Furthermore, the radiological tumor response after SIRT can be delayed, or due to tumor necrosis and edema, show initial pseudo progression followed by stable or responsive disease afterwards [25].

## 5. Conclusions

In conclusion, this is the first study to report the long-term liver-related outcome after SIRT in patients with HCC, and this study assessed the largest cohort of patients treated with SIRT to date with this endpoint in mind. Patients with intermediate- or advanced-stage HCC treated with SIRT have a substantial risk of developing liver decompensation, both in the short term (19% developed REILD) and in the longer term (>six months). Liver decompensation after SIRT is associated with shorter OS. Despite this risk, the survival of patients treated with SIRT who did not develop REILD was significantly better than matched patients treated with sorafenib. This suggests that, in well-selected patients, SIRT has a potential survival benefit over sorafenib. The ALBI score was predictive of both liver decompensation and OS and thus could be a valuable marker for patient selection.

## Figures and Tables

**Table 1:** Baseline, treatment and previous treatment characteristics of patients treated with SIRT.

Baseline Characteristics	Total Cohort (*n* = 85)	Study Cohort (*n* = 69)	REILD (*n* = 16)
Mean age in years (SD)	67.7 (8.5)	68.1 (8.4)	66.3 (9.4)
Sex, *n* (%)			
Male	73 (86)	61 (88)	12 (75)
Female	12 (14)	8 (12)	4 (25)
Comorbidities, *n* (%)			
None	27 (32)	25 (36)	2 (13)
Cardiovascular	30 (35)	22 (32)	8 (50)
Diabetes mellitus	32 (38)	24 (35)	8 (50)
Other	16 (19)	11 (16)	5 (31)
Etiology of liver disease, *n* (%)			
Alcohol	37 (44)	30 (44)	7 (44)
Hep B	10 (12)	8 (12)	2 (13)
Hep C	16 (19)	13 (19)	3 (19)
NAFLD	10 (12)	8 (12)	2 (13)
Unknown	10 (12)	7 (10)	3 (19)
Other	7 (8)	6 (9)	1 (6)
None	3 (4)	3 (4)	0 (0)
Cirrhosis, *n* (%)	62 (73)	49 (71)	13 (81)
Mean ALBI score (SD)	−2.7 (0.39)	−2.8 (0.37)	−2.6 (0.44)
ALBI grade, *n* (%)			
1	55 (65)	46 (67)	9 (56)
2	30 (35)	23 (33)	7 (44)
CP score, *n* (%)			
A5	73 (86)	61 (88)	12 (75)
A6	10 (12)	6 (9)	4 (25)
B7	2 (3)	2 (3)	0 (0)
Ascites, *n* (%)	9 (13)	9 (13)	0 (0)
Clinically irrelevant	6 (67)	6 (67)	N.A.
Mild	2 (22)	2 (22)	N.A.
Moderate-severe	1 (11)	1 (11)	N.A.
Portal hypertension, *n* (%)	42 (49)	31 (45)	11 (69)
MVI, *n* (%)	29 (34)	23 (33)	6 (38)
Tumor thrombus	26 (90)	21 (91)	5 (83)
Extra-hepatic metastasis, *n* (%)	6 (7)	6 (9)	0 (0)
BCLC stage, *n* (%)			
A	1 (1)	1 (1)	0 (0)
B	52 (61)	43 (62)	9 (56)
C	32 (38)	25 (36)	7 (44)
Prior treatment, *n* (%)	34 (40)	28 (41)	10 (63)
Resection	7 (8)	6 (9)	1 (6)
RFA	13 (15)	12 (17)	1 (6)
MWA	2 (2)	2 (3)	0 (0)
TACE	16 (19)	13 (19)	3 (19)
Sorafenib	6 (7)	5 (7)	1 (6)

BCLC: Barcelona clinic liver cancer; CP: Child-Pugh; Hep: hepatitis virus; MVI: macro vascular involvement; MWA: microwave ablation; N.A.: not applicable; NAFLD: non-alcoholic fatty liver disease; RFA: radio frequent ablation; SD: standard deviation; TACE: transarterial chemo embolization; TARE: transarterial radioembolization.

**Table 2:** Outcome summary of patients treated with SIRT.

Variable	Deceased Patients *n* = 45	Alive Patients *n* = 22
Survival after SIRT, median (95% CI) (months)	15 (95% CI 9.6–20.4)	20 (95% CI 16.8–23.3)
Cause of death, *n* (%)		
Tumor related	5 (11)	N.A.
Liver related	10 (22)	N.A.
Combined	18 (40)	N.A.
Unknown/Other	12 (27)	N.A.
At last follow-up		
Presence of progression, *n* (%)		
Yes	34 (76)	11 (50)
No	11 (24)	10 (45)
Missing	0 (0)	1 (5)
Time to progression, median (95% CI) (months)	6.0 (95% CI 5.6–6.4)	9.0 (95% CI 4.3–13.7)
Tumor response at last FU, *n* (%)		
Complete response	2 (4)	3 (14)
Partial response	9 (20)	4 (18)
Stable disease	4 (9)	8 (36)
Progressive disease	25 (56)	4 (18)
Could not be determined	3 (7)	3 (14)
Missing	2 (4)	0 (0)
Child-Pugh score at last FU, *n* (%)		
A5	5 (11)	10 (45)
A6	3 (7)	1 (5)
B7	6 (13)	5 (23)
B8	7 (16)	1 (5)
B9	8 (18)	1 (5)
C10	7 (16)	0 (0)
C11	1 (2)	0 (0)
C12	2 (4)	0 (0)
Missing	6 (13)	4 (18)
ALBI score at last FU, median (range)	−1.56 (−3.51–−0.10)	−2.47 (−3.44–−1.71)
ALBI grade at last FU, *n* (%)		
1	8 (18)	9 (41)
2	24 (53)	10 (46)
3	11 (24)	0 (0)
Missing	2 (4)	3 (14)
MELD at last FU, median (range)	14.50 (6–27)	8 (6–24)
Decompensation at last FU, *n* (%)		
Yes	31 (69)	7 (32)
No	8 (18)	11 (50)
Missing	6 (13)	4 (18)
Presence of ascites at last FU, *n* (%)		
Yes	29 (64)	6 (27)
No	13 (29)	16 (73)
Missing	3 (7)	0 (0)
Relevance of ascites at last FU, *n* (%)		
Clinically irrelevant	5 (17)	0 (0)
Mild	10 (34)	4 (67)
Moderate-severe	14 (48)	2 (33)

ALBI: albumin-bilirubin; 95% CI: 95% confidence interval; FU: follow-up; MELD: model for end-stage liver disease; N.A.: not applicable.

**Table 3:** Odds ratios for liver decompensation after SIRT (study cohort).

Variable	Odds Ratio	*n*	95% CI	*p*-Value
Gender	0.178	59	0.031–1.015	*p* = 0.085
Age	0.987	59	0.927–1.051	*p* = 0.688
Cirrhosis at baseline	**4.026**	**59**	**1.230** **–13.178**	***p* = 0.018**
Ascites at baseline	4.516	59	0.516–39.529	*p* = 0.238
Portal hypertension at baseline	2.222	59	0.733–6.733	*p* = 0.154
MELD score	0.981	42	0.630–1.261	*p* = 0.515
ALBI score	**0.074**	**59**	**0.012** **–0.475**	***p* = 0.006**

ALBI: albumin-bilirubin; 95% CI: 95% confidence interval; FIB-4: fibrosis-4; MELD: model for end-stage liver disease. *p* < 0.05 are in bold.

## Data Availability

The data that support the findings of this study are not publicly available due to privacy and ethical reasons, i.e., due to the fact that they contain information that could compromise the privacy of research participants, but are available from the corresponding author upon reasonable request.

## Supplementary Materials

The following are available online at https://www.mdpi.com/article/10.3390/cancers13215427/s1: Tables S1 and S3: Baseline characteristics for each study cohort; Tables S2 and S4: Post-matching characteristics.
