# Supplementary material for: Liver Decompensation as Late Complication in HCC Patients with Long-Term Response following Selective Internal Radiation Therapy
Source: Cancers (Basel). 2021 Oct 29;13(21):5427. doi: 10.3390/cancers13215427 (PMC8582376; doi:10.3390/cancers13215427)
Supplement: Supplementary file 1 [file cancers-13-05427-s001.zip › cancers-1390890-supplementary.pdf]

cancers MDPI

1. Definitions

Ascites was scored on a 4-point scale: absent, clinically irrelevant, mild and moderate-severe. Ascites was scored clinically irrelevant if it was observed on imaging but had no clinical implications (i.e. no medical intervention). Ascites was scored mild if patients expressed complaints and required medical intervention. Ascites was scored moderate-severe when on imaging great amounts of ascites were reported plus when diuretics did not result in resolving the ascites, or if paracentesis was needed.

Radiological signs of portal hypertension were defined as occurrence of splenomegaly, varices or collateral vein formation. Hepatic encephalopathy was scored by West-Haven criteria and was scored by the clinician who conducted the physical examination at follow-up.

2. Analyses 1: Total cohort SIRT vs sorafenib

Table S1. Baseline characteristics of each study cohort

	**Sorafenib(n=300)**	**SIRT (n=85)**
Age		
Mean (SD)	63.2 (10.0)	67.7 (8.54)
Median [Min. Max]	65.0 [26.0, 84.0]	68.0 [45.0, 84.0]
Sex		
Female	64 (21.3%)	12 (14.1%)
Male	236 (78.7%)	73 (85.9%)
Cirrhosis		
No	68 (22.7%)	23 (27.1%)
Yes	222 (74.0%)	62 (72.9%)
Missing	10 (3.3%)	0 (0%)
CP score		
A5	167 (55.7%)	73 (85.9%)
A6	46 (15.3%)	10 (11.8%)
B7	35 (11.7%)	2 (2.4%)
Missing	52 (17.3%)	0 (0%)
PH		
No	190 (63.3%)	43 (50.6%)
Yes	109 (36.3%)	42 (49.4%)
Missing	1 (0.3%)	0 (0%)
BCLC		
A	0 (0%)	1 (1.2%)
B	82 (27.3%)	52 (61.2%)
C	217 (72.3%)	32 (37.6%)
Missing	1 (0.3%)	0 (0%)
Extrahepatic disease		
No	127 (42.3%)	79 (82.9%)
Yes	172 (57.3%)	6 (7.1%)
Missing	1 (0.3%)	0 (0%)

Table S2. post matching characteristics.

	Sorafenib	SIRT
n=	76	76
sex = Male (%)	66 (86.8)	64 (84.2)
age (mean (SD))	66.21 (9.59)	67.49 (8.38)
cirrhosis = Yes (%)	60 (78.9)	56 (73.7)
CP score (%)		
A5	62 (81.6)	65 (85.5)
A6	12 (15.8)	9 (11.8)
B7	2 (2.6)	2 (2.6)
PH = Yes (%)	36 (47.4)	35 (46.1)
BCLC (%)		
1	0 (0.0)	0 (0.0)
2	45 (59.2)	45 (59.2)
3	31 (40.8)	31 (40.8)
Ext. Hep. Disease = Yes	5 (6.6)	6 (7.9)

3. Analyses 2: SIRT vs sorafenib liver decompensation

Table S3. Baseline characteristics of each study cohort

	**Sorafenib (n=198)**	**SIRT (n=69)**
Age		
Mean (SD)	63.8 (9.59)	68.1 (8.36)
Median [Min. Max]	65.0 [26.0, 84.0]	68.0 [50.0, 84.0]
Sex		
Female	39 (19.7%)	8 (11.6%)
Male	159 (80.3%)	61 (88.4%)
Cirrhosis		
No	50 (25.3%)	20 (29.0%)
Yes	141 (71.2%)	49 (71.0%)
Missing	7 (3.5%)	0 (0%)
CP score		
A5	115 (58.1%)	61 (88.4%)
A6	26 (13.1%)	6 (8.7%)
B7	17 (8.6%)	2 (2.9%)
Missing	40 (20.2%)	0 (0%)
PH		
No	132 (66.7%)	38 (55.1%)
Yes	65 (32.8%)	31 (44.9%)
Missing	1 (0.5%)	0 (0%)
BCLC		
A	0 (0%)	1 (1.4%)
B	56 (28.3%)	43 (62.3%)
C	141 (71.2%)	25 (36.2%)
Missing	1 (0.5%)	0 (0%)
Extrahepatic disease		
No	83 (41.9%)	63 (91.3%)
Yes	114 (57.6%)	6 (8.7%)
Missing	1 (0.5%)	0 (0%)

Table S4. Post matching characteristics.

	**Sorafenib**	**SIRT**
n=	55	55
sex = Male (%)	49 (89.1)	48 (87.3)
age (mean (SD))	66.02 (8.73)	67.36 (8.49)
cirrhosis = Yes (%)	40 (72.7)	39 (70.9)
CP score (%)		
A5	46 (83.6)	48 (87.3)
A6	7 (12.7)	5 (9.1)
B7	2 (3.6)	2 (3.6)
PH = Yes (%)	20 (36.4)	24 (43.6)
BCLC (%)		
1	0 (0.0)	0 (0.0)
2	33 (60.0)	36 (65.5)
3	22 (40.0)	19 (34.5)
Ext. Hep. Disease = Yes	5 (9.1)	5 (9.1)
